# *Lasiodiplodia syzygii* sp. nov. (Botryosphaeriaceae) causing post-harvest water-soaked brown lesions on *Syzygium samarangense* in Chiang Rai, Thailand

**DOI:** 10.3897/BDJ.9.e60604

**Published:** 2021-01-07

**Authors:** Chao-Rong Meng, Qian Zhang, Zai-Fu Yang, Kun Geng, Xiang-Yu Zeng, K. W. Thilini Chethana, Yong Wang

**Affiliations:** 1 Department of Plant Pathology, Agricultural College, Guizhou University, Guiyang, China Department of Plant Pathology, Agricultural College, Guizhou University Guiyang China; 2 Guiyang plant protection and inspection station, Guiyang, China Guiyang plant protection and inspection station Guiyang China; 3 Center of Excellence in Fungal Research, Mae Fah Luang University, Chiang Rai, Thailand Center of Excellence in Fungal Research, Mae Fah Luang University Chiang Rai Thailand; 4 School of Science, Mae Fah Luang University, Chiang Rai, Thailand School of Science, Mae Fah Luang University Chiang Rai Thailand

**Keywords:** Botryosphaeriaceae, fruit disease, new pathogen, wax apple

## Abstract

**Background:**

*Syzygium samarangense* (Wax apple) is an important tropical fruit tree with high economic and nutrient value and is widely planted in the tropics or subtropics of Asia. Post-harvest water-soaked brown lesions were observed on mature fruits of ornamental wax apples in Chiang Rai Province, Thailand. A fungus with morphological characters, similar to *Lasiodiplodia*, was consistently isolated from symptomatic fruits. Phylogenetic analyses, based on ITS, LSU, TEF1-a and *tub2*, revealed that our isolates were closely related to, but phylogenetically distinct from, *Lasiodiplodia rubropurpurea*.

**New information:**

Morphological comparisons indicated that pycnidia and conidiogenous cells of our strains were significantly larger than *L. rubropurpurea*. Comparisons of base-pair differences in the four loci confirmed that the species from wax apple was distinct from *L. rubropurpurea* and a new species, *L. syzygii* sp. nov., is introduced to accommodate it. Pathogenicity tests confirmed the newly-introduced species as the pathogen of this post-harvest water-soaked brown lesion disease on wax apples.

## Introduction

Wax apple [*Syzygium samarangense* (Blume) Merrill and Perry] belongs to the *Myrtaceae* and was naturalised in the Philippines thousands of years ago (Lim 2012, Shen et al. 2012). As a kind of juicy tropical fruit like watermelon with economic importance, it has been commonly and widely cultivated in many Asian countries (Nesa et al. 2014). Every part of *S. samarangense* also has potential medicinal values (Shen et al. 2012).

Due to the fruit characteristics, such as thin peel and tender pulp with high respiratory intensity, wax apples are prone to damage by pathogens and cannot be stored for a long time (Yang et al. 2009). This causes a significant post-harvest loss. Many studies suggest that wax apple is mainly threatened by fungal diseases. For example, a new fruit rot of wax apple caused by *Phytophthora palmivora* was reported in southern Taiwan during the rainy periods in 1982 (Lin et al. 1984). Yang et al. (2009) and Che et al. (2015) reported *Lasiodiplodia theobromae* as the causal agent of black spot disease on harvested wax apple fruits. *Pestalotiopsis samarangensis* was isolated from the fruit rot in wax apples from markets in Thailand (Maharachchikumbura et al. 2013). *Chrysoporthe deuterocubensis* caused cankers on wax apple and branches in Taiwan (Fan et al. 2013).

The present study reports a new post-harvest water-soaked brown lesion disease on wax apples caused by Lasiodiplodia sp. in Chiang Rai,Thailand. Morphological and multi-locus phylogenetic analyses revealed that our strain represented a novel species. A pathogenicity test on fruits confirmed the pathogenic relationship between *L. syzygii* and *Syzygium samarangense*.

## Materials and methods

### Sample collection, isolation and morphology

Rotten wax apple fruits were occasionally collected from a food market near Mae Fah Luang University in Chiang Rai, Thailand. On the third day after the wax apple fruits were collected, it was observed that there were conidiomata bulges on the surface of the fruit, white hyphae and the fruit turned black, rotted and had cytoplasmic extravasation. Diseased samples were conserved in self-sealing bags and then taken back to the laboratory and photographed. Before isolation, diseased fruits were surface disinfected with 70% ethanol for 30 s, 1% sodium hypochlorite (NaClO) for 1 min and repeatedly twice rinsed in sterile distilled water for 30 s. Pure cultures were obtained by single-conidium isolation following a modified method outlined by Chomnunti et al. (2011) and Maharachchikumbura et al. (2013). The morphology of fungal colonies was recorded following the method of Hu et al. (2007). Fungal mycelium and spores were observed under a light microscope and photographed. The holotype specimen is deposited in the Herbarium of the Department of Plant Pathology, Agricultural College, Guizhou University (HGUP). The ex-type and isotype cultures are deposited in the Culture Collection at the Department of Plant Pathology, Agriculture College, Guizhou University, P.R. China (GUCC) and the Mae Fah Luang University Culture Collection (MFLUCC) in Thailand.

### DNA extraction, PCR reaction and sequencing

Fungal cultures were grown on PDA at 28°C. When colonies nearly covered the entire Petri dish (90 mm diam.), fresh mycelia were scraped from the agar surface with sterilised scalpels. Genomic DNA was extracted using a BIOMIGA Fungus Genomic DNA Extraction Kit (GD2416) following the manufacturer’s protocol. DNA amplification was performed in a 25 μl reaction volume following Liang et al. (2018). Primers ITS1 and ITS4 (White et al. 1990) were used to amplify the internal transcribed spacer regions and intervening 5.8S rRNA region (ITS) and LR0R and LR5 for 28S rRNA (LSU) region (Vilgalys and Hester 1990, Rehner and Samuels 1994). Two protein-coding gene fragments, the β-tubulin (*tub2*) and translation elongation factor 1-alpha (TEF1-a) were amplified with primer pairs BT2A/BT2B (Glass and Donaldson 1995, O'Donnell and Cigelnik 1997) and EF1-688F/EF1-986R, respectively (Carbone and Kohn 1999, Alves et al. 2008). Purification and sequencing of the PCR amplicons were done by SinoGenoMax, Beijing. The DNA sequences are deposited in the GenBank and their accession numbers are provided in Table 1. The DNA base differences of the four loci amongst our strains and ex-type or representative strains of relative taxa are shown (Table 2).

### Phylogenetic analyses

Sequences of 45 *Lasiodiplodia* isolates, representing all species known from culture, were aligned using the online version of MAFFT v. 7.307 (Katoh and Standley 2016) and manually improved, where necessary, using MEGA v. 6.06 (Koichiro et al. 2013). Mesquite v. 2.75 (Maddison 2008) was used to concatenate the aligned sequences of the different loci. Ambiguous regions were excluded from analyses using AliView (Larsson 2014), gaps were treated as missing data and optimised manually with *Botryosphaeria dothidea* (CMW8000) and *B. fabicerciana* (CBS 127193) as the outgroups (Table 1). The alignment document has been deposited in TreeBASE (www.treebase.org) and the accession number is 27461. Phylogenetic analyses were constructed by Maximum Parsimony (MP), Maximum Likelihood (ML) and Bayesian Inference methods. First, the ambiguous regions were excluded from the alignment and gaps were treated as missing data. The MP analysis was done with PAUP v. 4.0b10 (Swofford 2002), using the heuristic search option with 1,000 random taxa addition and tree bisection and reconnection (TBR) as the branch swapping algorithm. Maxtrees was set to 5000. Tree length (TL), consistency index (CI), retention index (RI), rescaled consistency index (RC) and homoplasy index (HI) were calculated for each tree generated. The Maximum Likelihood (ML) analysis was performed using IQ-tree (Nguyen et al. 2015, Chernomor et al. 2016). Nucleotide substitution models were selected under the Akaike Information Criterion (AIC) by jModelTest2 (Darriba et al. 2012) on XSEDE in the CIPRES web portal (Miller et al. 2010). For the ITS dataset, the TPM3uf+I model was selected (-lnL = 1316.7068), for LSU, the TrN+I (-lnL = 1643.7273), for TEF1-a, the HKY+I+G (-lnL = 2399.0528) and for β-tubulin, the TIM3+G (-lnL = 1161.0392). ML was inferred under partitioned models. Non-parametric bootstrap analysis was implemented with 1000 replicates. Bayesian Inference (BI) analyses was conducted in MrBayes 3.2 (Ronquist et al. 2012). MrModeltest v.2.3 (Nylander 2004) was used to estimate the best evolutionary models under the Akaike Information Criterion (AIC). HKY+I was selected as the best model for ITS, for LSU, HKY+I+G, for TEF1-a, HKY+I+G and for β-tubulin, GTR+G was selected as the best model. Six Markov Chain Monte Carlo runs were launched with random starting trees for 1,000,000 generations and sampling every 1,000 generations. The first 25% resulting trees were discarded as burn-in.

### Pathogenicity tests

One isolate of the new *Lasiodiplodia* species (GUCC 9719.1) was grown on PDA and when the cultures covered the entire surface of the Petri dish, mycelia were scraped off with a sterilised blade. Conidiomata were crushed with a glass rod to prepare a spore suspension of 1× 10^5^ spores/ml. Pathogenicity testing was carried out on five healthy fruits of wax apple bought from the market. Inoculations were carried out in April 2020. The surface of the fruits was wiped with 70% ethanol and allowed to air-dry. Three fruits were slightly wounded by pin-pricking and 3 ml of spores suspension was sprayed on to the wound. The other two wounded fruits were maintained as control and inoculated with 2 ml of sterile deionised water. All inoculated fruits were placed in plastic bags, labelled and a high level of humidity was maintained for seven days by the addition of wet sterile cotton wool in each bag in an illuminated incubator at 28 ± 3°C. Daily observations were made on the development of disease symptoms. When fruits developed the symptoms, they were removed from the bags. Two isolates obtained from the diseased tissue were grown on PDA and then sequenced with primer pairs of the above four DNA markers to confirm the identity.

## Taxon treatments

### Lasiodiplodia syzygii

C.R. Meng, Qian Zhang & Yong Wang bis sp. nov.

36593BB5-E940-55FF-A752-C47223FD800D

837701

#### Materials

- **Type status:** Holotype. **Occurrence:** catalogNumber: HGUP 9719; recordedBy: Wang Yong; **Taxon:** scientificName: Lasiodiplodia syzygii; kingdom: Fungi; class: Dothideomycetes; order: Botryosphaeriales; family: Botryosphaeriaceae; genus: Lasiodiplodia; **Location:** country: Thailand; stateProvince: Chiang Rai; **Identification:** identifiedBy: Chao-Rong Meng; dateIdentified: 2020; **Record Level:** type: ex-type living culture GUCC 9719.1; MFLU 19-0565, isotype, isotype living culture MFLUCC 19-0257.
- **Type status:** Other material. **Occurrence:** catalogNumber: HGUP 9720 and HGUP 9721; recordedBy: Wang Yong; **Taxon:** scientificName: Lasiodiplodia syzygii; kingdom: Fungi; class: Dothideomycetes; order: Botryosphaeriales; family: Botryosphaeriaceae; genus: Lasiodiplodia; **Location:** country: China; stateProvince: Guiyang; **Identification:** identifiedBy: Chao-Rong Meng; dateIdentified: 2020; **Record Level:** type: living cultures GUCC 9719.2, GUCC 9719.3 and GUCC 9719.4

#### Description

*Pathogenic* on *Syzygium samarangense*. **Sexual morph**: Undetermined. **Asexual morph** (Fig. 2): *Conidiomata* up to 2 mm diam., pycnidial, covered with hyphae, black, globose, ostiolate, solitary, separate, uniloculate, immersed to semi-immersed. *Conidiomatal wall* composed of thick-walled, dark brown cells of *textura angularis*, becoming thin-walled and hyaline towards the inner region. *Paraphyses* cylindrical, aseptate, hyaline. *Conidiophores* reduced to conidiogenous cells. *Conidiogenous cells* 10–14.5 × 3.5–4.5 μm (average = 11 × 3.7 μm, n = 20), hyaline, smooth, holoblastic forming conidia at their tips. *Conidia* thick-walled, wall up to 1 μm wide, ovoid with both ends rounded, hyaline and remaining so for a long time, becoming pale brown with obsolete striations and occasionally with 1-septate after discharging from the conidioma, (27–)30–32(–36) × (13–)15–17(–20) μm (average = 31.3 × 16.4 μm, n = 50), L/W = 1.9.

*Culture characteristics*: Conidia germinate on PDA within 24 hours at room temperature (25–30°C) with germ tubes produced from both ends of the conidia. Colonies with white fluffy mycelium on PDA, after 7 days become olivaceous-grey at the centre, white at the edge, raised, fluffy, dense filamentous.

##### Notes

*Lasiodiplodia syzygii* strains are closely related to *L. rubropurpurea*, but formed a distinct, well-supported clade in the phylogenetic analyses. Base-pairs comparisons between *L. syzygii* ex-type strain (GUCC 9719.1) and ex-type strain of *L. rubropurpurea* (WAC 12535) found seven base differences (1.3%) in ITS region and five differences (0.6%) on LSU, but nine differences (2.1%) in *tub2* and 34 in TEF1-a (10.4%) (Table 2). *Lasiodiplodia syzygii* produced larger pycnidia (up to 2 mm) and larger conidiogenous cells (10–14.5 × 3.5–4.5 μm) than *L. rubropurpurea* (0.5–1.5 mm and 7–13 × 3–5 μm) (Burgess et al. 2006).

#### Etymology

In reference to the host from which the fungus was first isolated.

## Analysis

### Phylogenetic analyses

**Figure 1.**
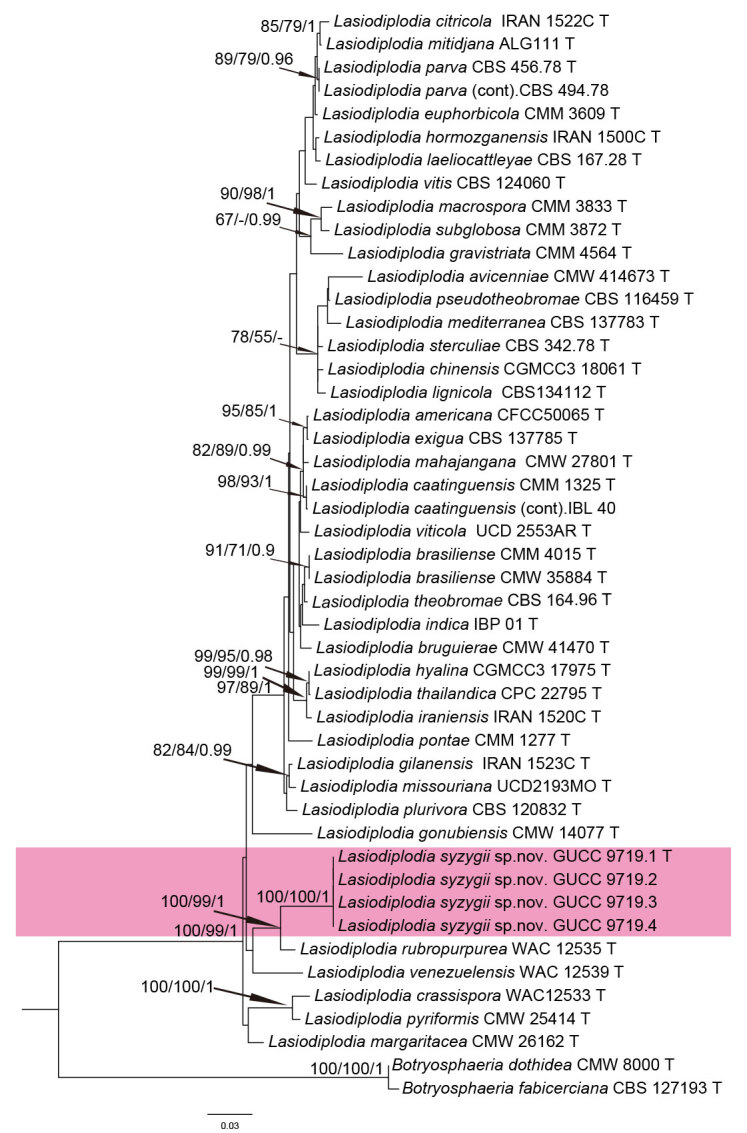
One of 850 most parsimonious trees obtained from a combined analyses of the ITS, LSU, TEF1-a and β-tubulin sequence dataset. Bootstrap values > 50% and BPP values > 0.90 are provided at the nodes and separated by “/”. Bootstrap values < 50% and Bayesian posterior probability (BPP) values < 0.90 were labelled with “-”. The tree was rooted with *Botryosphaeria fabicerciana* (CBS 127193) and *B. dothidea* (CMW 8000). The branch of the new *Lasidiodiplodia* species is highlighted with pink.

Four *Lasiodiplodia* strains isolated from *Syzygium samarangense* were sequenced. The final alignment of ITS, LSU, TEF1-a and *tub2* comprised of 2177 characters, viz. ITS: 1–530, LSU: 533–1423, TEF1-a: 1426–1752 and β-tubulin: 1755–2183. Of these, 1843 characters were constant and 73 were parsimony-uninformative. Maximum parsimony analysis of the remaining 261 parsimony-informative characters resulted in 850 most parsimonious trees (TL = 676, CI = 0.64, RI = 0.81, RC = 0.52 and HI = 0.36) and the first one is shown as Fig. 1. The ML and Bayesian analyses resulted in trees with similar topologies. Strains GUCC 9719.1, GUCC 9719.2, GUCC 9719.3 and GUCC 9719.4 formed an independent well-supported clade sister to Lasiodiplodia rubropurpurea (MP: 100%, ML: 100% and Bayesian posterior probability: 1) Comparison of the DNA base-pair differences between our strains and *L. rubropurpurea* species in four gene regions (Table 2) confirmed the presence of two species; therefore, a new species is introduced for those isolates from wax apple.

### Pathogenicity test on the fruits of wax apple

**Figure 2.**
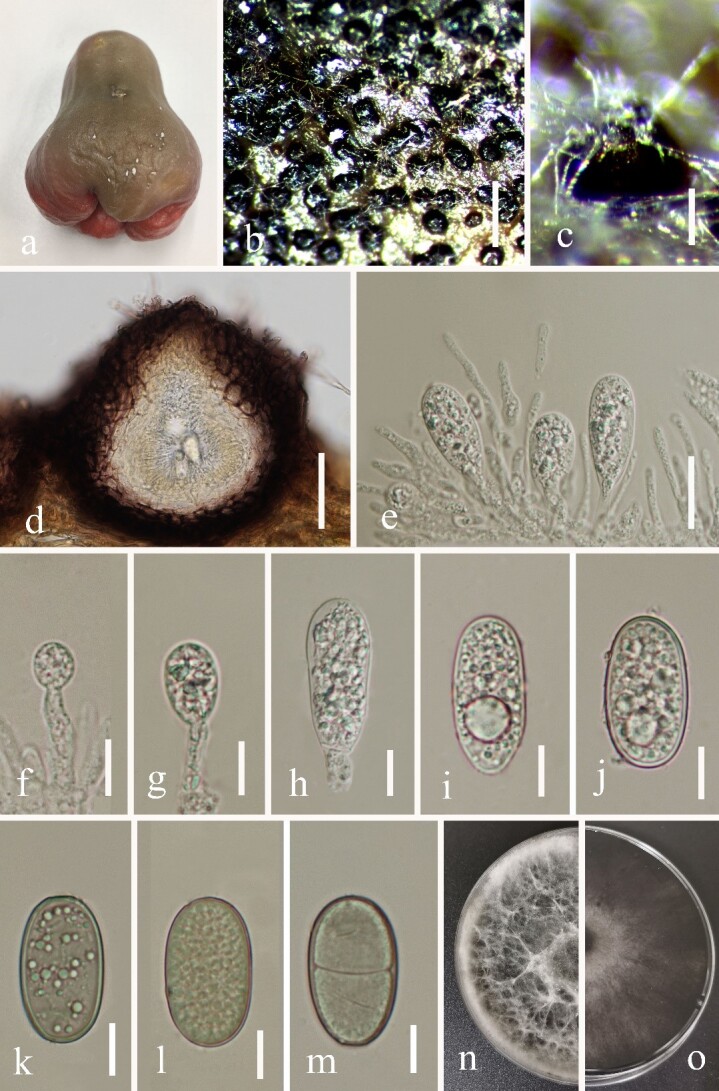
*Lasiodiplodia syzygii* (MFLUCC 19-0257). **a.** infected fruit; **b, c**. Conidiomata on the host; **d**. Section through a conidioma; **e.** Conidia developing amongst paraphyses; **f-h.** Conidia formed on conidiogenous cells; **i-m.** Immature conidia; **n-o.** Colonies on PDA culture; **n.** From above; **o.** From below. Scale bars: b = 300 μm, c = 140 μm, d = 50 μm, e = 20 μm, f–m = 10 μm.

**Figure 3.**
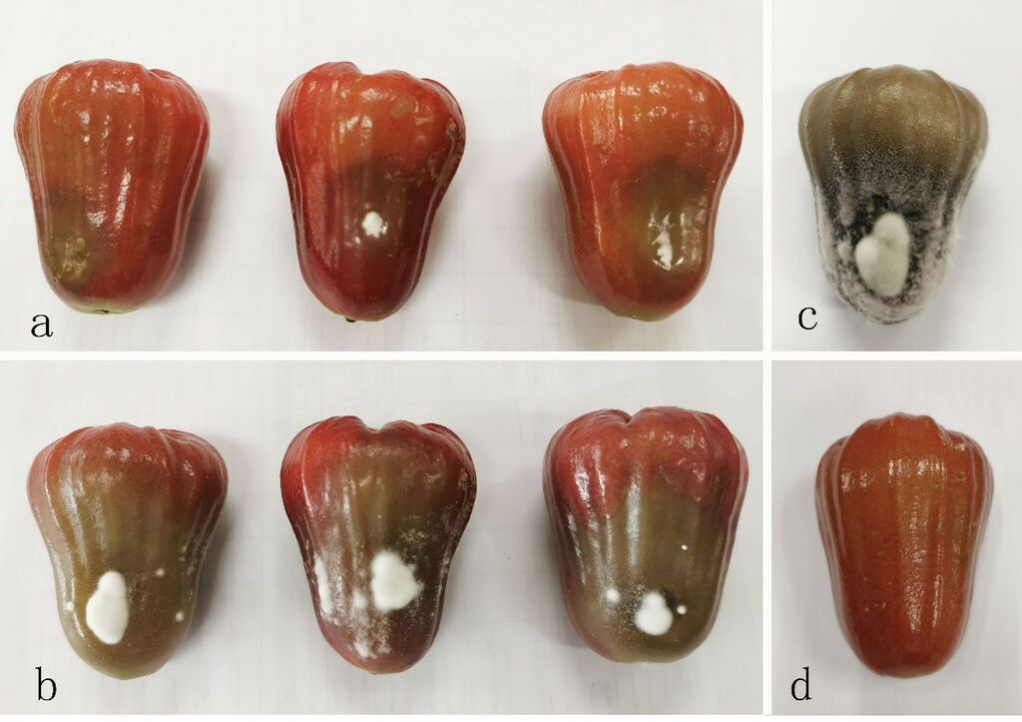
Symptoms developing on *Syzygium samarangense* fruits inoculated with *Lasiodiplodia syzygii*. **a.** Symptom at 3^rd^ day; **b.** Symptom at 5^th^ day; **c.** Symptom at 7^th^ day; **d.** Control.

At the third day after inoculation, water-soaked areas with a few white hyphae began to appear on all inoculated fruits similar to the naturally-infected wax apples (Fig. 2a and Fig. 3a). The water-soaked symptom of diffusion with abundant hyphae producing mycelium further appeared on inoculated *Syzygium samarangense* fruits after five days (Fig. 3b). At the 7th day after inoculation, the symptoms spread throughout the fruit (Fig. 3c), together with many white mycelia and more hyphae accompanied by cytoplasmic exosmosis. The control fruits (Fig. 3d) did not show any symptom. The fungi were re-isolated from the lesions of inoculated wax apple fruits and the re-identified (GUCC 9719.3 and GUCC 9719.4) sequencing four gene regions.

## Discussion

This study revealed a new species of *Lasiodiplodia*, *L. syzygi* from rotting fruits of *Syzygium samarangense*. Phylogenetic analyses, based on ITS, LSU, TEF1-a and *tub2*, showed that it is phylogenetically closer to *L. rubropurpurea*. Comparisons of DNA base-pair differences in the four loci, as well as morphological differences, confirmed the novelty of this species. The fungus was proved to be pathogenic and, therefore, it is the causal agent of the post-harvest water-soaked brown lesions on wax apple.

Wax apple (*Syzygium samarangense*) is known to be affected by many fungal pathogens that often cause economic losses. These include *Colletotrichum gloeosporioides* (Udayanga et al. 2013) and *Lasiodiplodia theobromae* which was the causal agent of black spot disease (Che et al. 2015), *Pestalotiopsis* spp. and *Phytophthora* spp. The fruit disease of the current study did not show any typical symptoms of black spot caused by *L. theobromae*. Furthermore, the pink or orange spore masses, typical of anthracnose caused by *C. gloeosporioides* or epidermal to superficial, acervular conidiomata reported by Maharachchikumbura et al. (2013) for *Pestalotiopsis*, were not seen in the current study. The fruit rot caused by *Phytophthora* spp. spread more rapidly (only 2 or 3 days up to a whole fruit) and results in a sour taste on fruits. However, the *L. syzygii* needed about seven days to completely rot the fruit and did not cause any sour taste in the fruits. Thus, the study reports a new disease on wax apple.

*Lasiodiplodia* resides in Botryosphaeriaceae, Botryosphaeriales (Hongsanan et al. 2020) and comprises several species known to cause important or potentially important diseases on woody hosts, mostly in the tropics or sub-tropics (Phillips et al. 2019). Very few species of this family appear to be host-specific (Dissanayake et al. 2016). In south-western China and adjoining areas, agriculture and forestry play an important role in the local economy, which might facilitate the spread of this wax apple disease. Thus, research needs to focus on the occurrence of this newly-discovered pathogen in other economically-important plants and in other locations, as well as how to manage it by biological or chemical control approaches. It is also remarkable to find a new disease on such an important commercial fruit indicating that there are numerous new taxa to be discovered in Thailand (Hyde et al. 2018) and Botryosphaeriaceae (Hyde et al. 2020).

## Supplementary Material

XML Treatment for Lasiodiplodia syzygii

## Figures and Tables

**Table 1.** **Table 1** GenBank accession numbers of isolates included in this study. Ex-type isolates are labelled with superscript T.

**Species**	**Isolate no.**	**GenBank no.**			
		**ITS**	**LSU**	***tef* 1**	***tub2***
*Lasiodiplodia americana*	CFCC50065^T^	KP217059	MF410052	KP217067	KP217075
*L. avicenniae*	CMW 414673^T^	KP860835	–	KP860680	KP860758
*L. brasiliense*	CMM 4015^T^	JX464063	–	JX464049	–
*L. brasiliense*	CMW 35884	KU887094	–	KU886972	KU887466
*L. bruguierae*	CMW 41470^T^	KP860833	–	KP860678	KP860756
*L. caatinguensis*	CMM 1325^T^	KT154760	–	KT008006	KT154767
*L. caatinguensis*	IBL 40	KT154762	–	KT154755	KT154769
*L. chinensis*	CGMCC3.18061^T^	KX499889	–	KX499927	KX500002
*L. citricola*	IRAN 1522C^T^	GU945354	–	GU945340	KU887505
*L. crassispora*	WAC12533^T^	DQ103550	DQ377901	EU673303	KU887506
*L. euphorbicola*	CMM 3609^T^	KF234543	–	KF226689	KF254926
*L. exigua*	CBS 137785^T^	KJ638317	–	KJ638336	KU887509
*L. gilanensis*	IRAN 1523C^T^	GU945351	–	GU945342	KU887511
*L. gonubiensis*	CMW 14077^T^	AY639595	DQ377902	DQ103566	DQ458860
*L. gravistriata*	CMM 4564^T^	KT250949	–	KT250950	–
*L. hormozganensis*	IRAN 1500C^T^	GU945355	–	GU945343	KU887515
*L. hyalina*	CGMCC3.17975^T^	KX499879	–	KX499917	KX499992
*L. indica*	IBP 01^T^	KM376151	–	–	–
*L. iraniensis*	IRAN 1520C^T^	GU945348	–	GU945336	KU887516
*L. laeliocattleyae*	CBS 167.28^T^	KU507487	DQ377892	KU507454	–
*L. lignicola*	CBS134112	JX646797	JX646814	KU887003	JX646845
*L. macrospora*	CMM 3833^T^	KF234557	–	KF226718	KF254941
*L. mahajangana*	CMW 27801^T^	FJ900595	–	FJ900641	FJ900630
*L. margaritacea*	CMW 26162^T^	EU144050	KX464354	EU144065	KU887520
*L. mediterranea*	CBS 137783^T^	KJ638312	–	KJ638331	KU887521
*L. missouriana*	UCD2193MO^T^	HQ288225	–	HQ288267	HQ288304
*L. mitidjana*	ALG111^T^	MN104115	–	MN159114	–
*L. parva*	CBS 456.78^T^	EF622083	KF766362	EF622063	KU887523
*L. parva*	CBS 494.78	EF622084	EU673258	EF622064	EU673114
*L. plurivora*	CBS 120832^T^	EF445362	KX464356	EF445395	KU887524
*L. pontae*	CMM 1277^T^	KT151794	–	KT151791	KT151797
*L. pseudotheobromae*	CBS 116459^T^	EF622077	EU673256	EF622057	EU673111
*L. pyriformis*	CMW 25414^T^	EU101307	–	EU101352	KU887527
*L. rubropurpurea*	WAC 12535^T^	DQ103553	DQ377903	DQ103571	EU673136
*L. sterculiae*	CBS 342.78^T^	KX464140	JX681073	KX464634	KX464908
*L. subglobosa*	CMM 3872^T^	KF234558	–	KF226721	KF254942
*L. syzygii*	MFLUCC 19-0219.1^T^	MT990531	MT990548	MW016943	MW014331
*L. syzygii*	GUCC 9719.2	MW081991	MW081988	MW087101	MW087104
*L. syzygii*	GUCC 9719.3	MW081992	MW081989	MW087102	MW087105
*L. syzygii* sp. nov.	GUCC 9719.4	MW081993	MW081990	MW087103	MW087106
*L. thailandica*	CPC 22795^T^	KJ193637	–	KJ193681	–
*L. theobromae*	CBS 164.96^T^	AY640255	EU673253	AY640258	KU887532
*L. venezuelensis*	WAC 12539^T^	DQ103547	DQ377904	DQ103568	KU887533
*L. viticola*	UCD 2553AR^T^	HQ288227	–	HQ288269	HQ288306
*L. vitis*	CBS 124060^T^	KX464148	KX464367	KX464642	KX464917
*Botryosphaeria dothidea*	CMW 8000^T^	AY236949	AY928047	AY236898	AY236927
*B. fabicerciana*	CBS 127193^T^	HQ332197	MF410028	HQ332213	KF779068

**Table 2.** DNA base pair differences between *Lasiodiplodia syzygii* and *L. rubropurpurea* in four separate loci. ^T^ = ex-type

*L. syzygiumae* strains	*Lasiodiplodia rubropurpurea* WAC 12535^T^			
	ITS (1–530)	LSU (531–1421)	TEF1-a(1422–1748)	*β*-tubulin (1749–2177)
MFLUCC 19-0257=GUCC 9719.1^T^	7	5	34	9
GUCC 9719.2	7	5	34	9
GUCC 9719.3	7	5	34	9
GUCC 9719.4	7	5	34	9
Total number of differences	55			
